# A Sick Nation

**Published:** 1862-09

**Authors:** 


### HALL'S JOURNAL OF HEALTH.

**Our Legitimate Scope is almost boundless: for whatever begets pleasurable and harmless feelings, promotes Health; and whatever induces disagreeable sensations, engenders Disease.**

WE AIM TO SHOW HOW DISEASE MAY BE AVOIDED, AND THAT IT IS BEST, WHEN SICKNESS COMES, TO TAKE NO MEDICINE WITHOUT CONSULTING A PHYSICIAN.

Vol. IX.] SEPTEMBER, 1862. [No. 9.

### A SICK NATION.

THE cause, the nature, and the remedy, are the three thoughts which always engage the attention of the scientific physician in every given case of sickness. Each individual may have his own opinion on these points, in reference to our country's present condition; and however obscure any man or woman may be, the expressed opinion of such person helps more or less to make up the great aggregate sentiment of the nation, and it is opinion which makes or destroys nationalities. Every patriot, in times of his country's trial, ought to sustain her to the extent of expressing his opinions in her favor; under still greater obligation is he laid, in proportion as he has an influence beyond himself. Hence the Editor here gives his opinion, pure and simple, and in as clear, sharp, and distinct terms as he knows how to express it: Maintain our republican institutions over the whole Union of States, if it require a lifetime's living on bread and water, wearing the coarsest home-spun, dwelling in huts if need be, selling every dollar's worth of property for the national treasury, and if required, leveling to the earth and clearing away, as clean as the palm of the hand, every city and city-site throughout the confederation, only if thereby the foundation is laid for the freedom of every slave that lives, and of every human being to be born within our boundaries, from this good hour. The scalpel which is handled less gingerly, had better be shivered to atoms before its employment. Men may philosophize and refine as much as they please, but clearly to our mind, the cause, the sole and only cause of this war, direct and remote, is slavery. The South saw with alarm that its power was waning, that its area was becoming more restricted, in the certain and prospective freedom of the border States, and in the growing population of the North-western territorial domain. They regarded the extension of the area of slavery as their salvation—and it was. The North was willing, in fidelity to the original understanding, to guarantee the South all their slave rights in its present boundary, but that one inch more of slave soil should be added to the national domain, was a moral impossibility. The South saw this, and the instinct of self-preservation impelled them to take the stand they did; it was an infinitely inevitable event, only a question of time.

The Bible declaration is the only safe ground on which to build in this thing, and in reference to any government whose laws emanate from the people, resistance to its authority is rebellion against God, for "he that resisteth the power, resisteth the ordinance of God;" and it is as utterly impossible for any nation to survive a rebellion against God, as it is for an individual to do so; hence the success of the South in its present undertaking to establish such a government as it proposes, is a moral impossibility. By analogy, they can never succeed. History is uniform in its teachings that the progress of empire and of domination is from the North, Southward. A law of the people may be a bad law, but for the time being, it is for all that to be recognized, until it can be changed by the dissemination of better views, and this is the only resistance which the Bible authorizes against legally constituted authority. Hence the only righteous alternatives are, submission to the laws of the land, peaceable removal, or the endurance of the legal penalty. Any other principle is subversive of all authority, and is anarchy in its worst form. With these views, we consider the doctrine of a "higher law" the "doctrine of devils;" and those who took this ground, and defended it, in reference to the fugitive slave law, were rebels, not only against the Government of the United States, but against God himself, and the South, in its conduct, has only followed Northern example; hence, whatever of vituperation and abuse and despicable epithet has been uttered, in speech or print, in reference to the Southern people, is only a degradation to those who uttered, or do utter them, for thereby they condemn themselves out of their own mouths. Besides, there is a want of magnanimity and dignity about it, which is unbecoming a man; it never can do good, and always will do harm. If the North must be an enemy to the South, let it be a high-toned, generous enemy, and not a hissing fiend, as some even of our clerical speakers have shown themselves to be. "Forgive them," was said in reference to the pitiless crucifiers. If there ever is a "higher law" than that which a people have framed for their own government, there is only one right course as to an existing law, either to obey it or submit peaceably to the penalty of its overt or passive infraction, even unto death, or leave the country.

The remedy, then, for the nation's malady is the perfect, utter and eternal eradication of the unfortunate cause, and that is the institution of slavery. But how apply the remedy? Go according to the law of the land, *as long as it is law;* free every slave instantly, belonging to a voluntary opposer of the Government of the United States; take from such persons all the property they have on earth; demand an unconditional oath of submission to the constituted authorities, or require them to leave the country forever, with the penalty of summary death if they return unbidden. As to the slaves of the loyal now living, let them be purchased at a fair price, and be set free; and all born hereafter, to be born free. What do with these millions? Do as Almighty wisdom did with Adam and Eve — turn them into the field of the world, and let them alone. Just give them as fair a chance as you would like any other human brother give to you. If, after that, you find some of them are likely to suffer, do as you would do to any stranger beggar — try and put them in a way of doing something for themselves. The negroes of the South have done a great deal toward the prosperity of this nation. Don't we owe them a little help? The gains which their sweat and toil have helped to make, have aided in building many a mansion in Fifth Avenue and Walnut street, on the Hudson and at Newport.

The man who does not stand by the President and his Cabinet as a unit, weakens them. Let every true man trust wholly to them, and sustain them by a generous confidence, in darkness as well as the light, for the very sufficient reason that they have all the lights of the case, and thus should be competent judges of the full situation, while they have at least as much patriotism and common-sense as any other equal number of persons in the land. A good many people seem to have just sense enough to believe that if the Government does not do as they think, all is lost; while the ubiquitous "our correspondent" of eight-by-ten sheets, declares about every other day there is a "crisis," and if that cannon on "point no point" is not directed a little further to the right or left by to-morrow at twelve o'clock, the nation will be remedilessly ruined. The Editor was born on slave soil, and lived among negroes for more than half his life, the remainder in the North, and the Southern people, as a class, rise before his mind daily as the personification of all that is cordial, high-minded, and heroic; and that what they have done in this war, with the means they had, has outshone the North in energy, self-sacrifice, endurance, and heroic daring, posterity will better testify; at the same time, that they are grievously in the wrong, and have committed a crime in this thing, second only to the crucifixion, we verily believe. But we know a great many good men and women all over the South, have personally known them from youth, and even infancy, and know that they never could have joined in this war unless they really believed it was right, and that it was their duty to do so. The North can not see how they can think it is right to subvert the best government the sun of heaven ever shone upon; neither can the South see why the North will not just let them alone; it was all they asked, it is all they ask to-day; so that neither side ought to be dictatorial, and both ought to be nobly forbearing.

At the same time, we are free to say that, under the circumstances of the case, the North ought to prosecute this war until the South shall surrender unconditionally — not to the North, but to the authority of the law of the land; that is the submission which the North ought to demand, ay, and the North will have it, if it costs the last dollar on the continent; and it ought to have it; and this contest ought no more to cease short of this end, and universal liberty, than the rebellion of Apollyon in heaven should have ceased short of his eternal overthrow. Human diseases allow no temporizing. One million of men ought to be drafted, and half of them on their way to the seat of war in thirty days, (the other half held in reserve,) without bounty, without advance payments, but on simple soldiers' wages; and then strike hard, strike fast, strike every where, and continuously, until the work of war is fully done, for, as the brave General Mitchel has significantly said, (brother Kentuckians, by the way, are we,) "We are engaged in war." Shall we employ the freed slaves, the rescued contrabands? Yes, in any way in which the laws of honorable warfare will allow. The very first and the most essential step in any man's elevation, is to let him see that he can do something, that he can help himself, that he can help others, that he can do a noble deed. After the war is over, the consciousness, "I have been a soldier," "I have striven side by side with the white man," will do more toward his elevation, in his own eyes, more toward sustaining his ambition, to keep him up where he is, and to rise higher, than five years of any other kind of teaching. One year in an active army, is ten at home. When we lived in New-Orleans, we often admired the manly bearing and the expression of self-respect and personal dignity which marked the countenances and conduct of the negroes who had fought with General Jackson on the fields of Chalmette, and we know, too, that those very negroes were always held in esteem—nay, that is not the full meaning, we will say in affectionate respect, by the old inhabitants of the Crescent City; and so are those who survive regarded by the oldest residents of New-Orleans this very hour. Still we would not arm the negro; let the whites have all the glory of the war, and victory. What next? *Since confiscation is the law of the land*, let all the confiscated soil be divided, by the Government, into conveniently small-sized parcels, and leased to any who will take them, for the cultivation of cotton, etc. If this is done, the annual yield of cotton, in five years, will approach eight million bales, instead of the four or five millions, some two years ago. We have often laughed in our sleeve, as many a cotton-planter has no doubt done, at the monstrously absurd admission of the North, that only negroes were fit to cultivate the burning plantations of the South, and that white men could not stand it. Dear, delightful ignoramuses of the North, did you ever inquire who dug the canals of the South, and ditched its millions of reclaimed lands? White men, for the most part. We have a medical student in our office now, who graduated with honor last spring at one of the best medical schools in the nation, who, at sixteen, made a full "hand" on his loyal father's immense plantation. We can give you the place and name and date and residence of families in the Gulf States, whose cotton was plowed and planted and hoed and gathered by the girls and boys, under the direction of the father, not a negro on the place; but they soon became able to own negroes, and now have plantations and "hands" of their own, all paid for. Having practiced medicine on Southern plantations, we speak by the authority of personal observation and actual knowledge of the facts.

If the South is thus dealt with, thus repopulated with working-men of any and all colors, it will, in the continuance of a rightly conducted republican government, by means of the elevating and restraining influences of a faithfully and intelligently preached Gospel, open up such a career of national prosperity, virtue, and greatness, as the sun never looked upon before, a government which will permanently hold in her own hands the balance of power for the world. But if this most unexpected and magnificent opportunity for universal liberty is not improved, then must such glorious results be indefinitely postponed, and the angels in heaven may well vail their faces before the throne of the great King, in sorrow and sadness unutterable. We are conscious that in what we have said we have offended our Southern friends, have offended all "Conservatives," as they like themselves styled, and we have offended "Union" men, whom we regard, with a few exceptions, as secessionists at heart, and now we are going to offend the "balance of the batch" of every body, by saying we would like to have the hanging of three men, the three abolition extremists, and feel like asking for one more, the anathemist, but he is a clergyman, and there are none of these to spare; besides, he has done work aforetime for the Sabbath and temperance, and other good causes, which is of silver and gold and precious stones, and for these, let him alone and compassionate him for the one great weak spot in his upper story. One hanging is not enough for these men. We would hang them every day in this wise: string them up by the big toe, nude, let a barrel of ice-water stream adown each, with daily birchings, and bread and water diet, until they learn to express themselves respectfully of God and man, of the Bible, our country, its rulers, its constitution, and its laws. The liberty of speech and of the press, is liberty no longer, but an impudent and vile prostitution of the same when they are employed, against legally constituted authority, in indecent allusions, in blasphemous exclamations, in degrading Billingsgate, and in fiend-like invective, shocking public sensibility and corrupting public taste, all of which have been but too common of late, in our public places and public prints.

In reference to the penalties which Congress has declared shall attach to men who shall persist, after September 25th, 1862, in refusing a true fealty to the best government which has ever been exercised on the face of the earth—namely, the loss of all their earthly substance—it is a just and righteous enactment; they have not only beggared multitudes who never harmed them, but have sent blood and death to thousands of happy homes, and have caused maimings to other thousands, which shall cause them in all after-life to live in destitution, from having been rendered incapable of labor, or to labor in ceaseless pain, and weary, wearing discouragement. Verily, many of them deserve to be banished from the land of their birth, to return no more, until they show by tears more bitter than wormwood, that they are fit subjects for the bestowment of a generous amelioration of penalties. Secession is not only a crime against our beloved country, but against our common humanity, inasmuch as its success is a blow to self-government, to republican institutions, to popular nationalities and true freedom throughout the world, which may not be recovered from for ages to come. If secession succeeds, then the United States becomes a fourth-rate power for centuries perhaps, to be obedient to the beck and call of kingdoms and empires beyond the seas, looking up to them as the eyes of a maiden look to the hand of her mistress. Rather than let our country come even within the shadow of perils like these, it would be better that every human being whose voice or heart or hand is continued to be raised against her in outright rebellion, or in the doubtful mask of "Union," of "conservatism," or of deprecating or halting adhesion to the authorities, better that all such should be swept from the face of the earth, that their memories rot, and their names be the synonyms of infamy, till time shall be no longer.

MONDAY, *July* 28*th*, 1862.

